# Adverse events associated with AstraZeneca COVID-19 vaccine among adults in Greater Kampala, Uganda: a cross-sectional study

**DOI:** 10.4314/ahs.v24i2.12

**Published:** 2024-06

**Authors:** Allan Komakech, Jonathan Izudi, John Kamulegeya, Freda L Aceng, James Acaye, Edirisa Juniour Nsubuga, Petranilla Nakamya, Daniel Kadobera, Lilian Bulage, Benon Kwesiga, Alex R Ario

**Affiliations:** 1 Uganda National Institute of Public Health, Kampala, Uganda; 2 Department of Community Health, Faculty of Medicine, Mbarara University of Science and Technology; 3 Data Synergy and Evaluations, African Population and Health Research Center, Nairobi, Kenya; 4 World Health Organization, Kampala, Uganda; 5 Department of Integrated Epidemiology, Surveillance and Public Health Emergencies, Ministry of Health, Kampala, Uganda; 6 Clarke International University, Kampala, Uganda; 7 Mulago National Referral Hospital, Kampala, Uganda

**Keywords:** Adverse events, assessment, COVID-19, Greater Kampala, Uganda

## Abstract

**Background:**

Uganda started AstraZeneca COVID-19 vaccination in March 2021 but information about adverse events is limited. We assessed adverse events following AstraZeneca vaccination among adults in Greater Kampala, Uganda.

**Methods:**

In this cross-sectional study, we systematically sampled persons who had received ≥1 dose of the AstraZeneca vaccine and collected data between March and April 2021 through telephone interviews. We defined adverse events as any untoward medical occurrence after vaccination and serious adverse events as any event leading to hospitalization, persistent disability >28 days, death, or congenital anomaly.

**Results:**

Of 374 participants aged 20-85 years, the prevalence of adverse events was 76.5%. Common adverse events included injection site redness and hadache; no serious adverse event was reported. Participants aged 20–29 years (Adjusted odds ratio (AOR) 4.58; 95% confidence interval (CI): 1.92–10.95), 30-39 years (AOR 3.69; 95% CI: 1.81–7.51) and 40-49 years (AOR 2.78; 95% CI 1.26–4.90) were more likely to develop adverse events compared to those aged ≥50 years.

**Conclusion:**

Adverse events are prevalent, largely among those aged <50 years; serious adverse events are rare. Persons aged <50 years should be targeted for surveillance of adverse events alongside appropriate health education and counselling.

## Background

On March 11, 2020, the World Health Organization (WHO) declared Coronavirus Disease of 2019 (COVID-19) a pandemic^1^. To slow the spread of severe acute respiratory coronavirus 2 (SARS-CoV-2), the virus which causes COVID-19, different public health preventive and control measures were implemented globally. These measures included social distancing, partial and comprehensive lockdowns, closure of schools, businesses, and places of social amenities besides enforcement of mandatory wearing of face masks in public places^2^. These measures initially flattened the epidemic curve but the resurgence of COVID-19 was reported as societies and economies re-opened^3^. Accordingly, vaccination of the population was advanced as a long-term preventive measure to address the COVID-19 morbidity and mortality in different countries^4^. The first vaccines were approved in November 2020 by WHO^5^ and since then, several vaccines have been rolled out in different countries, with at least 200 additional vaccine candidates under development^6^. The commonly used vaccines include AstraZeneca, Moderna, BioNTech, Pfizer, Johnson & Johnson, Sinopharm, and Gamaleya vaccines^7^.

Although vaccines confer a protective effect against COVID-19, they are not without side effects. Studies report that adverse events can occur within seconds to weeks following vaccination^8^. However, the adverse events, either serious or non-serious, may not necessarily have a causal relationship with the use of the vaccine^8^,^9^. The serious adverse events result in death, hospitalization, permanent/persistent disability, and congenital anomalies/defects among others while the non-serious ones do not lead to any of these health events^10^,^11^.

In Uganda, the Ministry of Health (MoH) started vaccination with the AstraZeneca vaccine in March 2021 in all the 136 districts in the country^12^, initially targeting teachers, health workers, security personnel, humanitarian front-line workers, persons aged ≥50 years, and those aged 18–49 years with comorbidities^13^ as high-risk populations for COVID-19. Adverse events are tracked through an active surveillance system by the MoH following the WHO recommendations, largely in the post-authorization or emergency use listing period^14^. Tracking of adverse events provides data about vaccine safety at the population level. The known adverse events for the AstraZeneca COVID-19 vaccine include injection site events such as pain, redness and swelling, headaches, fever, and malaise among others^15^. However, additional concerns about serious adverse events such as deaths, clots, and severe allergic reactions have been raised^16^. However, the Uganda MoH and WHO indicate that the AstraZeneca vaccine is safe and effective against COVID-19^17^. Despite the nationwide rollout of the AstraZeneca vaccine in Uganda, there is a paucity of data about the magnitude of adverse events following the vaccination with the AstraZeneca vaccine. To fill this gap, we determined the prevalence of adverse events following the AstraZeneca vaccination, characterized the adverse events, and determined the factors associated with adverse events among adults in Kampala, Uganda. This information is important in tracking the safety of the AstraZeneca COVID-19 vaccine among the Ugandan adult population and possibly beyond.

## Methods and materials

### Study design and setting

We conducted a cross-sectional study in the Greater Kampala district, which is made up of Kampala, Wakiso, and Mukono districts. The study was conducted between March 10, 2021, and April 30, 2021. These districts had the highest proportion of individuals that had received the AstraZeneca COVID-19 vaccine at the time, with Kampala at 15%, Mukono at 2%, and Wakiso at 1.7%. Kampala had five sites designated for COVID-19 vaccination in each of its five divisions, a total of 25 vaccination sites. Wakiso and Mukono districts had five vaccination sites each.

### Study population and sampling

Our study population consisted of adults who had received at least one dose of the AstraZeneca COVID-19 vaccine. Individuals who did not have their phone contacts or contacts of their next of kin were excluded from the study because they could not be reached for interviews. At the vaccination sites, we used probability proportionate to the size of vaccinated individuals to determine the number of participants for the study. At the time of the data collection, approximately 36,000 individuals had received the AstraZeneca vaccine in Kampala, Wakiso, and Mukono combined. We apportioned our sample size among the 35 vaccination sites based on the number that had received the vaccine. We used a systematic sampling with a random start to select the participants from the study sites of Kampala, Mukono, and Wakiso districts.

### Study variables and data collection

We used a standardized questionnaire adapted from the WHO core variables for adverse events (9) to collect the data through phone interviews. We collected data on age, sex, nationality, profession, chronic illness, history of previous reactions to a vaccine, and the details of adverse events among those reporting to have experienced adverse events, namely the type of adverse events experienced, the dose after which the adverse event had occurred, time of onset of adverse event and the duration, and the outcome of the adverse event. In the event of non-response for reasons of the phone being switched off, unreachable, or poor network connectivity amongst others, we attempted to contact the eligible participants twice, two days apart and thereafter we contacted the participants' next of kin. Eligible participants that remained unreachable were considered non-responders.

The primary outcome was adverse events following AstraZeneca COVID-19 vaccination measured on a binary scale (yes versus no) defined as any untoward medical occurrence after vaccination. Any adverse event that resulted in hospitalization, death, a permanent/persistent disability, a congenital anomaly, or was considered life-threatening in an individual who had received ≥1 dose of AstraZeneca COVID-19 vaccine was considered a serious adverse event and all the rest were considered as a non-serious adverse event.

### Quality control measures

Prior to the data collection, we trained the interviewers on the questionnaire, and interviewing techniques. We also pretested the tools to address any potential ambiguities and provided detailed instructions and protocols to ensure uniformity in the data collection process. After the data collection, respondents with mssing data were replaced with other respondents to ensure no missing variables in our data. All completed questionnaires were reviewed for completeness before the data entry. Quality checks like skip patterns, alerts, legal values, and range values were applied during data entry. We also randomly sampled the entered questionnaires and checked then for accuracy of the data entries.

### Statistical issues

#### Sample size estimation

The sample size was determined using the Kish Leslie (1964) formula. The assumptions included a 95% confidence interval, 50% estimated incidence of adverse events, and a margin of error (precision) of 0.05. We estimated that 384 participants were required and inflated this number by 15% to account for non-response. The final sample size for this study was estimated at 442 participants.

#### Data analysis

Data were entered into a Microsoft word Excel sheet and analyzed using Statistical Package for the Social Sciences (SPSS) software version 24. We descriptively summarized the data using frequencies and percentages and presented them using tables and graphs. We performed bivariate analysis to establish differences in the outcome with independent variables using the Chi-square test for large cell counts and Fisher's exact test for small cell counts. All variables with probability values (p-value) of < 0.05 were fitted in a logistic regression model to establish those independently associated with the outcome. Variables with p-values <0.05 at multivariable analysis were considered statistically significant. The results were stated as crude odds ratio (COR) and adjusted odds ratio (AOR).

### Ethical consideration

This study was performed in response to a public health emergency and was, therefore, determined to be a non-research assessment. The Uganda MoH provided the directive to conduct the assessment and the Office of the Associate Director for Science, U.S Centers for Disease Control and Prevention (U.S CDC), Uganda, equally determined that the undertaking was not human subject research since the primary intent was public health practice. All the protocols for this project were reviewed in line with the CDC policy by the US CDC human subjects review board in accordance with the Declaration of Helsinki. Verbal informed consent in the local languages was sought from the participants before the data were collected. We used a verbal mode of consent as the participants were contacted on phone and therefore written consent was not possible. This procedure of verbal consent was approved by the US CDC human subjects review board. All methods were carried out in accordance with relevant guidelines and regulations.

## Results

### Demographic and clinical characteristics of participants

Of the required 442 participants, we studied 374 participants giving a response rate of 84.6%. The mean age of the participants was 41 ± 13.0 years and ranged from 20 years to 85 years, with 198 (52.9%) males making up slightly more than half of the participants (Table 1). Overall, the study showed that 286 (76.5%) participants had experienced adverse events after AstraZeneca COVID-19 vaccination, with systematic differences observed in adverse events concerning age (p= <0.001), sex (p= 0.040), and currently on long-term medication (p=0.003).

**Table 1:** Demographic and clinical characteristics of participants

		Adverse event		
Variables	Overall	Yes	No	P-value
	No. (%)	No. (%)	No. (%)	
**District**				0.111
Kampala	307 (82.1)	241 (84.3)	66 (75.0)	
Mukono	36 (9.6)	23 (8.0)	13 (14.8)	
Wakiso	31 (8.3)	22 (7.7)	9 (10.2)	
**Age group (years)**				<0.001*
20–29	73 (19.5)	64 (22.4)	9 (10.2)	
30–30	113 (30.2)	95 (33.2)	18 (20.5)	
40–49	90 (24.1)	71 (24.8)	19 (21.6)	
≥50	98 (26.2)	56 (19.6)	42 (47.7)	
**Sex**				0.040*
Male	198 (52.9)	143 (50.0)	55 (62.5)	
Female	176 (47.1)	143 (50.0)	33 (37.5)	
**Nationality**				0.134
Ugandan	365 (97.6)	281 (98.3)	84 (95.5)	
Non-Ugandan	9 (2.4)	5 (1.7)	4 (4.5)	
**Have chronic illness**				0.116
Yes	104 (27.8)	73 (25.5)	30 (34.1)	
No	270 (72.2)	213 (74.5)	58 (65.9)	
**Currently on any long-term medication**				0.003*
Yes	70 (18.7)	44 (15.4)	62 (70.5)	
No	304 (81.3)	242 (84.6)	26 (29.5)	
**Ever had previous reactions to vaccinations**				0.351
Yes	19 (5.1)	17 (5.9)	2 (2.3)	
No	330 (88.2)	251 (87.8)	79 (89.8)	
Not sure	25 (6.7)	18 (6.3)	7 (7.9)	
**Usually have reactions to any medicine such as antibiotics, anti-inflammatories**				0.697
Yes	47 (12.6)	37 (12.9)	10 (11.4)	
No	327 (87.4)	249 (87.1)	78 (88.6)	
**Illness at the time you received the COVID-19 vaccine**				0.354
Yes	22 (5.9)	40 (14.0)	7 (8.0)	
No	352 (94.1)	246 (86.0)	81 (92.0)	

* p-value<0.05

### Adverse event after the first AstraZeneca COVID-19 vaccine dose

**Figure 1:**
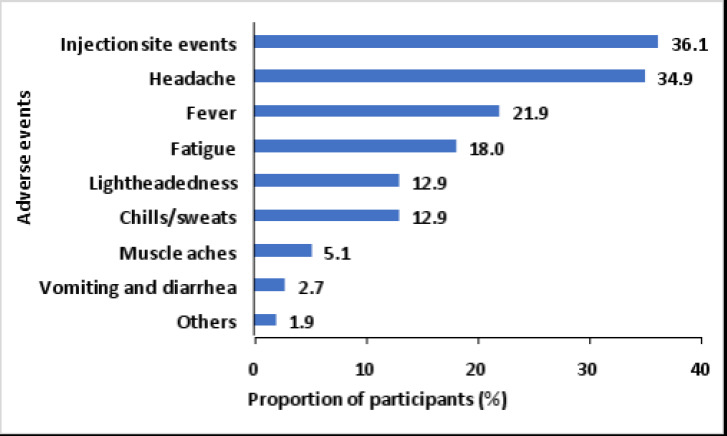
Adverse events following the first dose of AstraZeneca vaccine in Greater Kampala, Uganda

Following the first dose of the AstraZeneca vaccine (Figure 1), most (36.1%) of the participants experienced injection site events, followed by headaches (34.9%), fever (21.9%), muscle aches (5.1%), and vomiting and diarrhea (2.7%).

### Adverse event after the second AstraZeneca COVID-19 vaccine dose

**Figure 2:**
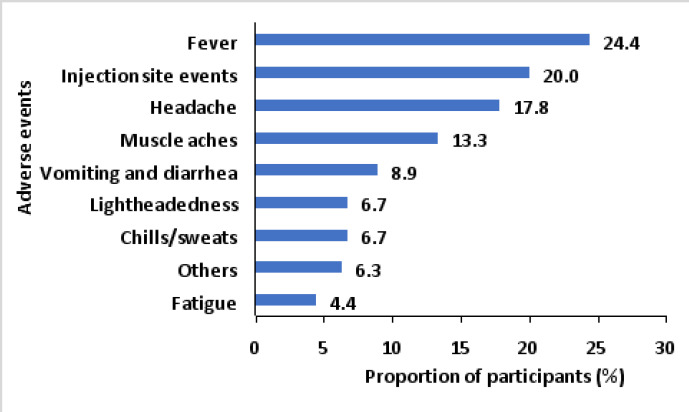
Adverse events following the second dose of AstraZeneca COVID-19 vaccination in Greater Kampala, Uganda

After the second dose of the AstraZeneca vaccine (Figure 2), most (24.4%) of the participants experienced fever, followed by injection site events (20.0%) and headache (17.8%); the least experienced adverse event was fatigue (4.4%).

### Time of onset of adverse events following vaccination with AstraZeneca vaccine

**Figure 3:**
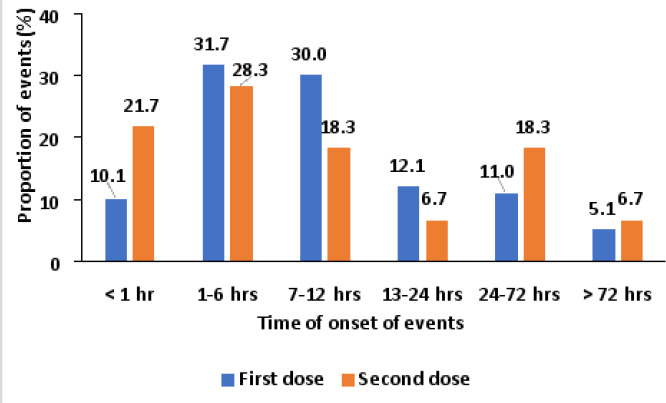
Time of onset of adverse events following vaccination with AstraZeneca COVID-19 vaccine, Greater Kampala, Uganda

Most (31.7%) of the adverse events after the first dose commenced within 1-6 hours, followed by 7-12 hours (30.0%) and less than 1 hour (10.1%). The least (5.1%) proportion of events commenced more than 72 hours after vaccination (Figure 3). Following the second dose, most (28.3%) of the events commenced within 1-6 hours, followed by within an hour (21.7%) and the least (6.7%) was within 13-24 hours or >72 hours (Figure 3).

### Outcome of adverse events following vaccination with AstraZeneca COVID-19 vaccination in Greater Kampala, Uganda

**Figure 4:**
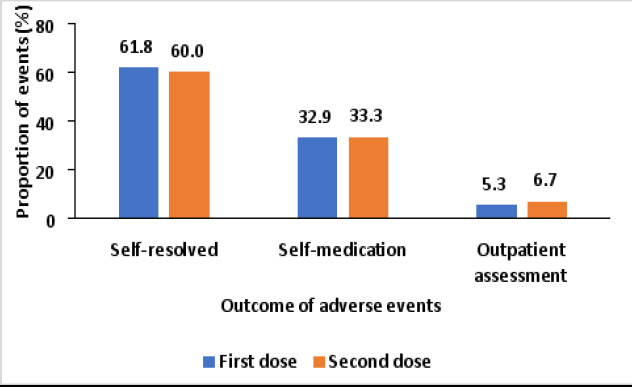
Outcome of adverse events following AstraZeneca COVID-19 vaccination in Kampala, Uganda

Figure 4 shows that most (61.8%) adverse events following the first dose were self-resolved, with some requiring self-medication (32.9%) and the least (5.3%) resulting in outpatient assessment. Similarly, following the second dose, most (60.0%) events self-resolved, followed by those requiring self-medication (33.3%) and the least (6.7%) resulting in outpatient assessment.

### Factors associated with adverse events following AstraZeneca COVID-19 vaccination

Table 2 summarizes the logistic regression analysis findings and shows that age, sex, and current long-term medication use were associated with an adverse event. In the univariable logistic regression analysis, participants aged 20-29 years (Crude odds ratio (COR) 5.33, 95% CI, 2.39-11.92), 30-39 years (COR 3.96, 95% CI 2.08-7.53), and 40-49 years (COR 2.80, 95% CI, 1.47-5.34) were more likely to develop an adverse event compared to those aged ≥50 years (Table 2). Females were more likely to develop an adverse event compared to males (COR 1.67, 95% CI, 1.02-2.72), and participants on long-term medication were less likely to develop an adverse event compared to those not on long-term medication (COR 0.43, 95% CI, 0.25-0.76).

**Table 2:** Factors associated with adverse events following AstraZeneca COVID-19 vaccination in Greater Kampala, Uganda

Variables	Adverse event, n (%)	No adverse event, n (%)	COR(95% CI)	AOR(95% CI)
**District**				
Kampala	241 (84.3)	66 (75.0)	1.49(0.66-3.40)	1.65(0.70-4.08)
Mukono	23 (8.0)	13 (14.8)	0.72(0.26-2.03)	0.86(0.27-2.73)
Wakiso	22 (7.7)	9 (10.2)	1	1
				
**Age group (years)**				
20–29	64 (22.4)	9 (10.2)	5.33(2.39–11.92)*	4.58(1.92–10.95)*
30–39	95 (33.2)	18 (20.5)	3.96(2.08–7.53)*	3.69(1.81–7.51)*
40–49	71 (24.8)	19 (21.6)	2.80(1.47–5.34)*	2.78(1.26–4.90)*
≥50	56 (19.6)	42 (47.7)	1	1
**Sex**				
Male	143 (50.0)	55 (62.5)	1.0	1.0
Female	143 (50.0)	33 (37.5)	1.67(1.02–2.72)*	1.53(0.91–2.59)
**Nationality**				
Non–Ugandan	281 (98.3)	84 (95.5)	1	1
Ugandan	5 (1.7)	4 (4.5)	0.37(0.10–1.42)	0.52(0.12–2.17)
**Have chronic illnesses**				
Yes	73 (25.5)	30 (34.1)	1	1
No	213 (74.5)	58 (65.9)	0.68(0.41–1.15)	0.44(0.15–1.25)
**Currently on any long-term medication**				
Yes	44 (15.4)	62 (70.5)	1	1
No	242 (84.6)	26 (29.5)	0.43(0.25–0.76)*	0.34(0.11–1.04)
**Ever had previous reactions to vaccinations**				
Yes	17 (5.9)	2 (2.3)	1	1
No	251 (87.8)	79 (89.8)	0.37(0.09–1.65)	0.65(0.14–3.03)
Not sure	18 (6.3)	7 (7.9)	0.30(0.06–1.67)	0.54(0.10–3.31)
**Usually have reactions to any medicine**				
Yes	37 (12.9)	10 (11.4)	1	1
No	249 (87.1)	78 (88.6)	0.86(0.41–1.82)	0.69(0.30–1.59)
**Illness at the time you received the COVID-19 vaccine**				
Yes	40 (14.0)	7 (8.0)	1	1
No	246 (86.0)	81 (92.0)	1.56(0.61-3.96)	1.08(0.35–3.39)

Note: COR= Crude Odds Ratio, AOR= Adjusted Odds Ratio

* indicates p<0.05

After controlling for confounders such as chronic illnesses, previous reactions to vaccines and medicines, and acute illness at the time of vaccine reception, participants aged 20-29 years (Adjusted odds ratio (AOr) 4.58; 95% CI: 1.92–10.95), 30-39 years (AOR 3.69; 95% CI: 1.81–7.51) and 40-49 years (AOR 2.78; 95% CI 1.26–4.90) were more likely to develop an adverse event compared to those ≥50 years old (Table 2). Being a female (AOR 1.53; 95% CI: 0.91-2.53) and currently on long-term medication [AOR 0.34; 95% CI: 0.11-1.04) were not associated with an adverse event.

## Discussion

Our study focused on adverse events following vaccination with the AstraZeneca vaccine. The study shows that at least seven in 10 people experience an adverse event following either the first or second dose of the vaccine, with the most common adverse events related to the injection site, headache, and fever mostly within 3 days of vaccination and lasting 1-3 days. Our data show persons aged 20–29 years, 30-39 years and 40-49 years are more likely to develop adverse events compared to those aged ≥50 years. Overall, we did not find any serious adverse events related to the AstraZeneca COVID-19 vaccine.

Most participants in our study revealed that they experienced an adverse event after either the first or the second dose of the vaccine. Our findings might be explained by AstraZeneca vaccine-induced immune response which is consistent with existing evidence as vaccines are known to induce an immune response 18–20,20,21. In a previous prospective study in Ethiopia, more than six in 10 healthcare providers vaccinated with AstraZeneca experienced adverse events^22^. Therefore, our findings are not unique. In Togo, at least seven in 10 people vaccinated with AstraZeneca reported more than one adverse event consistent with the present findings. In Bangladesh, evidence from a cross-sectional study showed that 51% of the participants vaccinated with AstraZeneca had experienced an adverse event^23^. Overall, there is sufficient evidence to show that adverse events following vaccination with AstraZeneca are common and our findings add to this body of literature. The differences in the proportion of adverse events might be attributable to differences in study setting, study population, and approach to data collection. We used telephone interviews which might be undermined by participants not being able to freely express themselves. However, the proportion of adverse events we report is not any different from those reported for other COVID-19 vaccines. For example, Pfizer–BioNTech COVID-19 vaccine (BNT162b2) is reported to have a 65% prevalence of adverse events while Johnson and Johnson vaccine has an 80% prevalence of adverse events^24^. There is a possibility that the differences in the prevalence of adverse events might be explained by ethnicity according to a previous study^25^.

We found that the most reported adverse events included those related to the injection site, fever, headaches, and general body weakness, which is consistent with studies conducted elsewhere^24^,^26^–^28^. These events might be explained by vaccine immune stimulation resulting in increased blood flow at the injection site hence raised body temperature. As the majority of the adverse events are expected, our findings imply a need to sensitize recipients of the AstraZeneca vaccine about these adverse events and the training of health workers to manage the adverse events if and when they occur.

No serious adverse events were reported in our study. This could be because the symptoms are short-lived or self-limiting and tend to be mild or moderate in severity. Most of the adverse events were self-resolved and none was a serious adverse event consistent with a study in Ethiopia^22^ and elsewhere^29^,^30^.

We found that participants aged 20–29 years, 30-39 years, and 40-49 years were more likely to develop adverse events compared to those aged ≥50 years. This might be explained by the degree of inflammatory-induced reactions between young and older persons. Young people tend to have more pronounced inflammatory-induced reactions following vaccination compared to older people leading to adverse events. Also, compared to younger people, the immune system in older persons is weaker hence older people do not have marked inflammatory-induced immune response. This lessens their risk of adverse events 31. Older persons might not report adverse events regarding them as symptoms of ageing^32^. Our finding of a more likelihood of the adverse event in young people compared to older people is consistent with several studies^22^,^27^,^30^,^33^.

## Strengths and limitations of the study

To the best of our knowledge, this is the first study in Uganda to assess adverse events following the AstraZeneca COVID-19 vaccine. We used a standardized tool developed by the WHO to assess adverse events and this makes our study replicable. However, there are some limitations which should be considered in the results interpretation. Although we report no serious adverse events following vaccination with AstraZeneca vaccine in this population, these adverse events would have best been studied using a longitudinal study design, which will allow a long time for follow-up. Our response rate was 84.6%, which is relatively high considering that online surveys have low response rate^34^. However, there is a possibility that the non-response might have been more in a particular demographic group than others to the extent that it might have influenced the outcome of interest. Our outcome is common, so the odds ratio overestimated the degree of association. Prevalence risk ratio would have been the preferred measure of effect. However, both measures would provide same direction of association although varying magnitudes. Adverse events were based on self-reporting so there is a possibility of social desirability or recall bias. Our sample size is relatively small so there is a possibility that the study might have been underpowered to detect a statistically significant effect on the population. Our study was conducted in an urban setting so the findings might not generalize to a rural setting. Despite these limitations, our data provide credible evidence for the immunization program in Uganda and beyond.

## Conclusion

Our study shows that adverse events are prevalent among persons vaccinated with the AstraZeneca vaccine. However, we found no serious adverse events. The commonest adverse events include those related to the injection site, headaches, and fever. People below the age of 50 years are more likely to experience an adverse event compared to those ≥50 years. Largely, the AstraZeneca COVID-19 vaccine is safe. Nonetheless, persons <50 years old should be targeted for surveillance of adverse events and receive appropriate health education and counseling.

## Data Availability

The datasets used and/or analysed during the current study belong to the Ministry of Health Uganda and are not publicly available. However, the data can be availed from the corresponding author upon reasonable request and with permission from the Ministry of Health Uganda.

## List of Abbreviations

AOR: Adjusted odds ratio
CDC: U.S Centers for Disease Control and Prevention
COR: Crude odds ratio
COVID-19: Coronavirus Disease 2019
MoH: Ministry of Health
SARS-CoV-2: Severe Acute Respiratory Syndrome Coronavirus 2
UNEPI: Uganda National Expanded Program on Immunization
WHO: World Health Organization
